# Delayed Hearing Recovery After Transverse Temporal Bone Fracture with Otic Capsule violation - Case Report and Literature Review

**DOI:** 10.1007/s12070-023-04145-x

**Published:** 2023-08-29

**Authors:** Magdalena Ostrowska, Anitta Sisily Joseph, Maciej J. Wróbel

**Affiliations:** Department of Otolaryngology, Collegium Medicum Nicolaus Copernicus University, Ul. Marie Sklodowskiej-Curie 9, Bydgoszcz, 85-094 Poland

**Keywords:** Sensorineural hearing loss, Tinnitus, Balance, Inner ear, BAHA

## Abstract

We present a patient who suffered a temporal bone fracture (TBF) encompassing the bony labyrinth. Sensorineural hearing loss was confirmed with an unfavorable prognosis for recovery. Thirteen years later, there is regression of the hearing loss.

## Introduction

Temporal bone fracture (TBF) usually occurs as a result of a high-energy blunt trauma to the head [1]. The classical division of fractures is based to the course of the fracture fissure in relation to the long axis of the temporal bone pyramid: longitudinal (L TBF), transverse (T TBF) and mixed (M TBF). The latest classification considers the presence or absence of labyrinth damage: otic capsule violation fractures (OCV) and otic capsule sparing fractures (OCS) [2–4]. Former, result in severe dysfunction of the vestibular and cochlear parts of the inner ear. In about 50% of cases, TBF causes immediate sensorineural hearing loss (SNHL) and severe dizziness. In the case of damage to the fluid containing spaces of the bony labyrinth and disturbances of fluid homeostasis, the damage is permanent [1]. With that in mind, authors present a case report of the patient, initially diagnosed with unilateral profound SNHL and severe vestibular disfunction after a transverse TBF OCV, in whom auditory perception improved years after the injury.

## Case Report

**Fig. 1:**
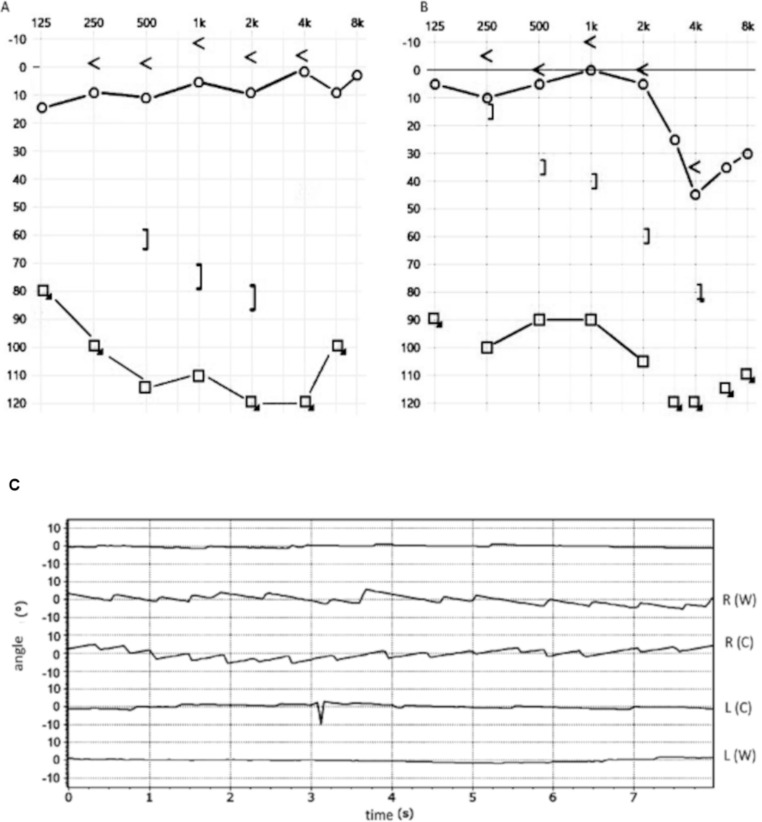
Additionally tests. Pure tone audiometry obtained (**a**) 4 months after injury, (**b**) 13 years after; < = bone conduction right ear; ] = bone conduction left ear; ○ = air conduction right ear; □= air conduction left ear and (**c**) videonystagmography - caloric test with Fitzgerald Hallpike’s modification – water temperature: 30 and 44^o^C, irrigation time 20s; R(W) – right ear/44^o^C; R(C) – right ear/30^o^C; L(W) – left ear/44^o^C, L(C) – left ear/30^o^C

A 20-year-old patient was admitted to Intensive Care Unit due to injuries from motorcycle accident. The following was observed: bleeding from the mouth, nose and left external auditory canal. Computed tomography (CT) imaging revealed multi-fragmentary fractures of the skull, including a TBF. The fracture fissure ran transversely across long axis of the temporal bone pyramid and encompassed the bony labyrinth. The function of the facial nerve was preserved. The patient was treated conservatively. Three months after, otoneurologic diagnostics were performed (PTA, speech audiometry, BERA, ENG / VNG). Left-sided severe SNHL and persistent tinnitus were confirmed. A control examination performed 4 months later confirmed the initial diagnosis (Fig. 1a). In VNG examination, left-sided uncompensated vestibular hypofunction was found. In light of the all findings, the patient’s prognosis for hearing recovery was unfavorable. Thirteen years after, Weber’s test revealed lateralization to the ear diagnosed as deaf. The subsequent PTA showed mixed hearing loss in the left ear with a bone conduction threshold of 10 dB (250 Hz) down to 80dB (4000 Hz) and air conduction thresholds of 90 dB at 125 Hz to 120 dB (4000 Hz) (Fig. 1b).

**Fig. 2:**
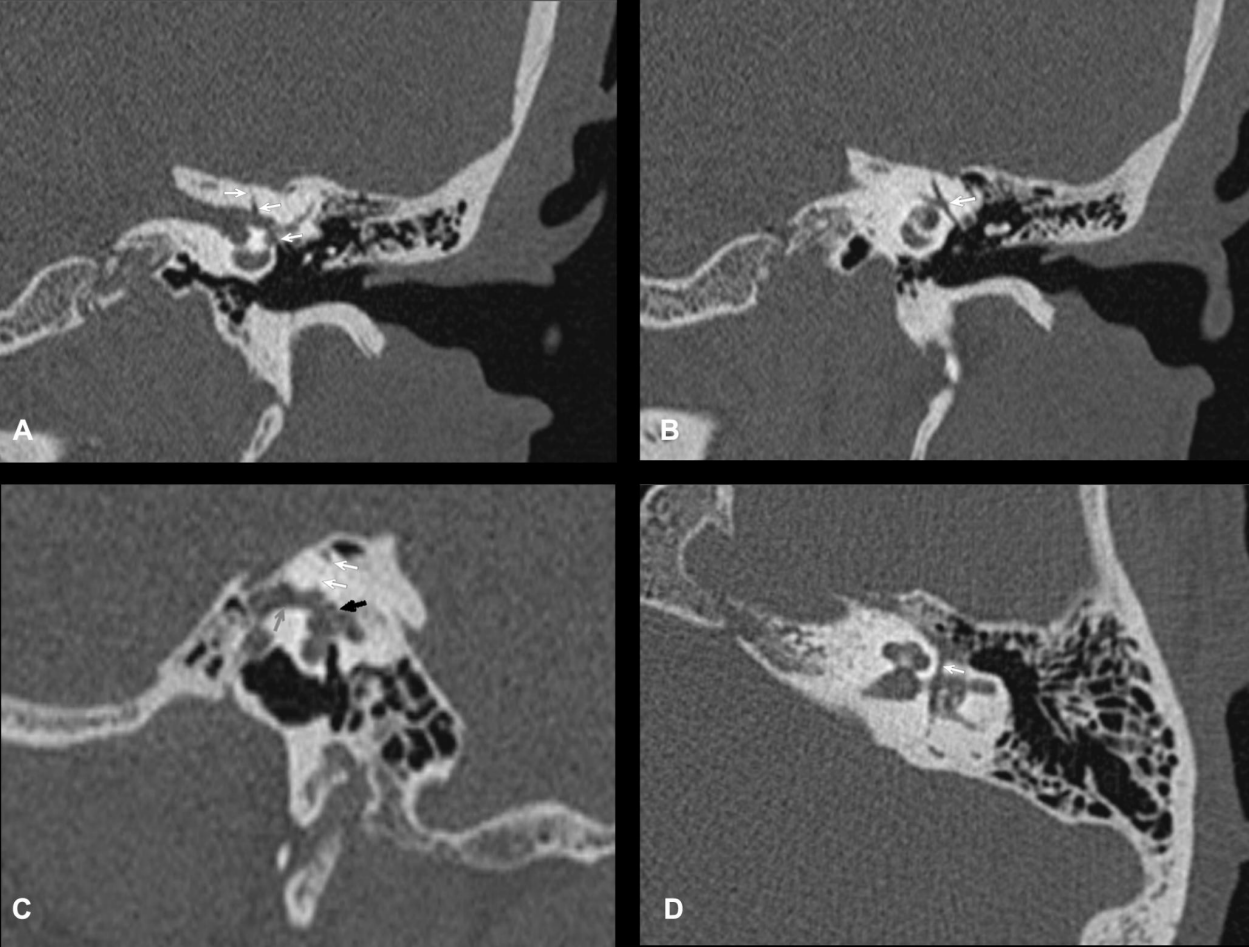
CT scans of the left inner ear. (**A**) coronal plane, white arrows present fracture affecting vestibule and internal acoustic meatus; (**B**) coronal plane, white arrows present the fracture, which is in close vicinity of the cochlea but is not damaging it; (**C**) sagittal plane, white arrows points at fracture, gray arrow points at internal acoustic meatus, black arrow: vestibule; (**D**) axial plane, fracture involves the vestibule

CT scans confirmed fracture of the pyramid, with scar tissue within the epitympanum, in direct contact with the fracture fissure (Fig. 2). Due to the risk associated with the of opening of the labyrinth, the patient wasn’t suitable for tympanoplasty. Bone conduction device was proposed instead, which was accepted by the patient.

Simultaneously, the peripheral vestibular system was reassessed (VNG, static posturography, cVEMP). The VNG tests showed: left-sided vestibular hypofunction (caloric test with Fitzgerald Hallpike’s modification), spontaneous nystagmus without fixation to the right side and positional nystagmus to the right (Nylen II) (Fig. 1c). The cVEMP study revealed a reduction in the left side wave amplitude, which significantly correlates with the result of the caloric test. The index of lateral vestibular coordination (posturography) was distorted. The tests confirmed permanent damage to the vestibular organ. The patient didn’t report any symptoms related to balance disturbances.

## Discussion

Only 5% of all TBFs are associated with damage of the bony labyrinth (OCV). The most common symptoms due to these fractures are: profound SNHL, paralysis of the facial nerve, CSF leakage, and dizziness [5]. In such cases, the prognosis for full recovery, especially hearing, is very unfavorable [1, 6]. A study by H. Kanon et al. including 815 TBFs showed a 7-fold higher likelihood of SNHL in fractures involving the bony labyrinth than in the labyrinth-sparing fractures. Profound hearing loss or deafness was demonstrated in 50% of OCV cases [5], whereas in the remaining cases, 3.8% presented with mild hearing loss, 26.9% - moderate, and 19.2% - severe. Brodie and Thompson demonstrated profound hearing loss in all subjects in their analysis of 21 OCV cases. Despite the limited literature on this subject, spontaneous regression of the hearing loss resulting from such an injury wasn’t confirmed [4].

In this case, the patient wasn’t able to determine precisely when hearing improved. Audiologic examination 13-years after the TBF revealed hearing loss with air-bone gap.

Dizziness and a feeling of instability after injuries of the temporal bone occur in 23–81% of cases in the first days of trauma [7]. These problems usually disappear within days or weeks, but there are cases when the imbalance is permanent [8]. Patient didn’t report any vestibular complaints since trauma. The initial VNG test revealed uncompensated weakening of the excitability of the left labyrinth. The control examination documented the hypofunction of the left labyrinth with no compensation mechanisms (spontaneous nystagmus without fixation, positional one-direction nystagmus, asymmetric rotational chair test). The otoneurologic test results without clinical correlation, indicate incorrect central compensation which may be related to the trauma and damage to the central nervous system [9]. In the largest available study of natural remission of post-traumatic symptoms (hearing loss and dizziness) in a group of 26 patients with OCV, it was found that after 3 weeks, 25 out of 26 patients had vestibular injury symptoms, but hearing loss was unchanged from the baseline examination after the injury. In a follow-up of 2–7 years, no clinical improvement was found in terms of hearing loss as compared to the assessment after 3 weeks after the injury [6]. Ricciardiello et al. in their analysis showed in all cases of TBFs (n = 30) in which SNHL was noted, it was irreversible [10].

## Conclusions

Although the mechanism is unknown, there is a chance of delayed hearing recovery in a damaged labyrinth. Despite the absence of vestibular complaints, the vestibular tests performed after temporal bone injury prove to be justified. They allow the monitoring of vestibular compensation or may suggest central nervous system disturbances. Therefore, it is suggested to maintain audiological and vestibular follow-up in such patients even years after trauma.
